# Aqua­{2-[(3,5-dichloro-2-oxidobenzyl­idene)amino]-3-(4-hydroxy­phen­yl)propionato-κ^3^ *O* ^1^,*N*,*O* ^2^}copper(II) sesquihydrate

**DOI:** 10.1107/S1600536808012038

**Published:** 2008-05-03

**Authors:** Zheng Liu, Yong Liao Wang, Li Wang

**Affiliations:** a Key Laboratory of Non-ferrous Metal Materials and Processing Technology, Department of Materials and Chemical Engineering, Guilin University of Technology, Ministry of Education, Guilin 541004, People’s Republic of China

## Abstract

In the title compound, [Cu(C_16_H_11_Cl_2_NO_4_)(H_2_O)]·1.5H_2_O, the Cu^II^ atom is coordinated by two O atoms and one N atom from the 2-[(3,5-dichloro-2-oxidobenzyl­idene)amino]-3-(4-hydroxy­phen­yl)propionate ligand, and by the O atom from a water mol­ecule in a square-planar coordination. There are two formula units in the asymmetric unit. Molecules are further assembled into a three-dimensional network through C—H⋯Cl contacts, a Cu⋯Cl weak inter­action [3.161 (2) Å], O—H⋯O  and C—H⋯O hydrogen bonds. The three water mol­ecules of the asymmetric unit are distributed over five positions with one full and two approximately half occupancies, while a tyrosine side chain in one of the complex mol­ecules is disordered over two positions [occupancies 0.507 (5) and 0.493 (5)].

## Related literature

For related literature, see: Casella & Gullotti (1986 ▶); Guthrie *et al.* (1980 ▶); Wang *et al.* (1994 ▶); Zhang *et al.* (2003 ▶).
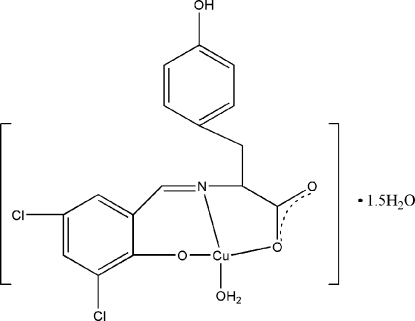


## Experimental

### 

#### Crystal data

[Cu(C_16_H_11_Cl_2_NO_4_)(H_2_O)]·1.5H_2_O
*M* *_r_* = 460.74
Triclinic, 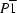

*a* = 10.006 (2) Å
*b* = 13.899 (2) Å
*c* = 14.565 (2) Å
α = 70.469 (2)°
β = 87.427 (3)°
γ = 73.121 (3)°
*V* = 1823.8 (5) Å^3^
*Z* = 4
Mo *K*α radiation
μ = 1.53 mm^−1^
*T* = 293 (2) K
0.40 × 0.18 × 0.09 mm

#### Data collection

Bruker SMART 1000 diffractometer
Absorption correction: multi-scan (*SADABS*; Sheldrick, 1996 ▶) *T*_min_ = 0.580, *T*_max_ = 0.875
9183 measured reflections
6315 independent reflections
3605 reflections with *I* > 2σ(*I*)
*R*_int_ = 0.027

#### Refinement

*R*[*F* ^2^ > 2σ(*F* ^2^)] = 0.047
*wR*(*F* ^2^) = 0.143
*S* = 1.02
6315 reflections
583 parameters
H-atom parameters constrained
Δρ_max_ = 0.58 e Å^−3^
Δρ_min_ = −0.36 e Å^−3^

### 

Data collection: *SMART* (Bruker, 2004 ▶); cell refinement: *SAINT* (Bruker, 2004 ▶); data reduction: *SAINT*; program(s) used to solve structure: *SHELXS97* (Sheldrick, 2008 ▶); program(s) used to refine structure: *SHELXL97* (Sheldrick, 2008 ▶); molecular graphics: *SHELXTL* (Sheldrick, 2008 ▶); software used to prepare material for publication: *SHELXTL*.

## Supplementary Material

Crystal structure: contains datablocks global, I. DOI: 10.1107/S1600536808012038/cs2072sup1.cif

Structure factors: contains datablocks I. DOI: 10.1107/S1600536808012038/cs2072Isup2.hkl

Additional supplementary materials:  crystallographic information; 3D view; checkCIF report

## Figures and Tables

**Table 1:** Hydrogen-bond geometry (Å, °)

*D*—H⋯*A*	*D*—H	H⋯*A*	*D*⋯*A*	*D*—H⋯*A*
O3—H3⋯O12^i^	0.82	1.88	2.643 (10)	154
O8—H8⋯O7^ii^	0.82	1.70	2.515 (9)	175
O5—H33⋯O6^iii^	0.85	1.89	2.714 (6)	164
O13—H42⋯Cl1^iv^	0.85	2.77	3.555 (9)	154
C26—H26⋯O3^v^	0.93	2.44	3.365 (7)	173

Symmetry codes: (i) 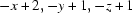; (ii) 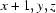; (iii) 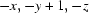; (iv) 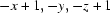; (v) 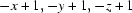.

**Fig. 1.**
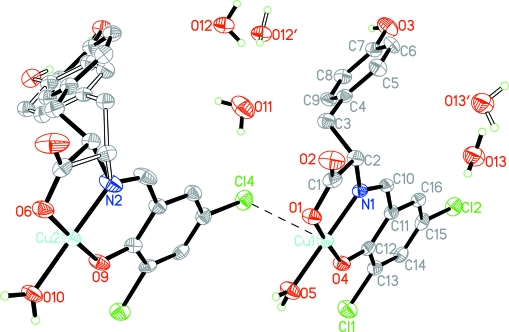
A view of the title complex showing 30% probability displacement ellipsoids. H atoms, except for the water molecules, are not shown.

**Fig. 2.**
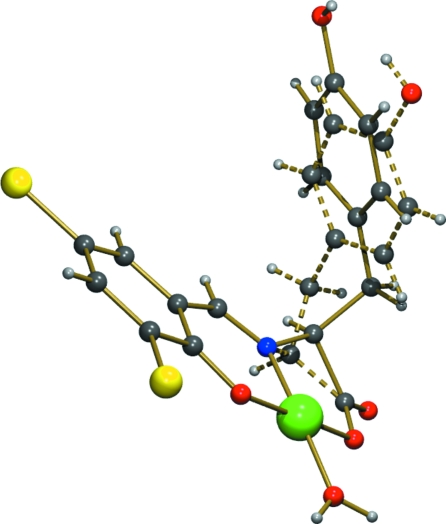
The disordered tyrosine view in one of the complexes.

**Fig. 3.**
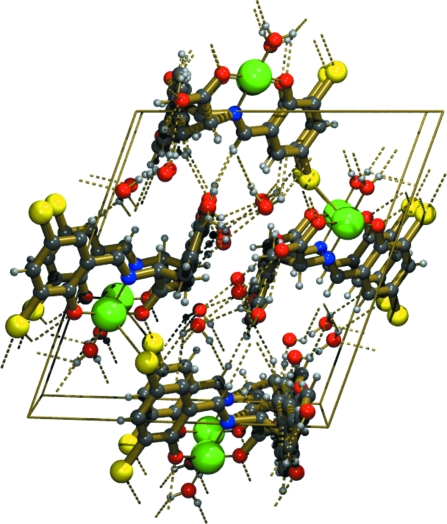
The three-dimensional network of Cu···Cl weak interactions and hydrogen bonds shown in dotted lines.

## supplementary crystallographic information

### Comment

Schiff base complexes containing amino acids have been studied for many years(Casella & Gullotti, 1986; Wang *et al.*, 1994; Zhang *et al.*, 2003). Schiff bases have played an important role in the development of coordination chemistry as they form stable complexes with most of the transition metals.

In the asymmetric unit there are two Cu^II^ ions, two *L* ligand anions, two ligating water molecules and three water hydrate molecules (Fig. 1). The Cu^II^ cations are coordinated by two O atoms and one N atom from ligand *L*, then the O atom from an H_2_O coordinate to Cu^II^. These four atoms form a square-planar coordination centered at the cuprate(II) ions (Fig. 1). The coordination in these two complexes in the asymmetric unit does not seem to be alike. There is a Cu···Cl distance of 3.161 (2) Å indicating a possible weak interaction between the two complexes in the asymmetric unit. Fig. 2 shows that the tyrosine in one of the complexes has two different orientations in this crystal giving a disordered structure. Weak contacts of C–H···Cl and Cu···Cl types and O–H···O hydrogen bonds construct a 3-D network running along the *a* axis (Fig.3).

### Experimental

An ethanol solution (5 ml) containing 3,5-dichloro-2-hydroxy-benzaldehyde (0.096 g, 0.5 mmol) was added to an aqueous solution containing 2-amino-3-(4-hydroxy-phenyl)-propionic acid (0.222 g, 2 mmol) and sodium hydroxide (0.091 g, 0.5 mmol). After stirring for 1 h, an aqueous solution of copper chloride (0.1 g, 0.5 mmol) was added to the resulting solution and stirred for 2 h. The green solution was filtered. After 10 days, green block crystals were obtained by slow evaporation of the filtrate (yield: 45.2%, based on Cu).

### Refinement

Water H atoms were located in a difference Fourier map and were allowed to ride on the O atom, with *U*_iso_(H) = 1.5*U*_eq_(O). All other H atoms were positioned geometrically and refined as riding, with C–H = 0.93 Å and with *U*_iso_(H) = 1.2 *U*_eq_(C).

### Crystal data

**Table:**

[Cu(C_16_H_11_Cl_2_N_1_O_4_)(H_2_O_1_)]·1.5H_2_O_1_	*Z* = 4
*M**_r_* = 460.74	*F*_000_ = 936
Triclinic, *P*1	*D*_x_ = 1.678 Mg m^−^^3^
*a* = 10.006 (2) Å	Mo *K*α radiation λ = 0.71073 Å
*b* = 13.899 (2) Å	Cell parameters from 2332 reflections
*c* = 14.565 (2) Å	θ = 2.3–25.2º
α = 70.469 (2)º	µ = 1.53 mm^−^^1^
β = 87.427 (3)º	*T* = 293 (2) K
γ = 73.121 (3)º	Plate, green
*V* = 1823.8 (5) Å^3^	0.40 × 0.18 × 0.09 mm

### Data collection

**Table:**

Bruker SMART 1000 diffractometer	6315 independent reflections
Radiation source: fine-focus sealed tube	3605 reflections with *I* > 2σ(*I*)
Monochromator: graphite	*R*_int_ = 0.027
*T* = 293(2) K	θ_max_ = 25.0º
φ and ω scans	θ_min_ = 1.5º
Absorption correction: multi-scan(SADABS; Sheldrick, 1996)	*h* = −11→11
*T*_min_ = 0.580, *T*_max_ = 0.875	*k* = −12→16
9183 measured reflections	*l* = −16→17

### Refinement

**Table:**

Refinement on *F*^2^	Secondary atom site location: difference Fourier map
Least-squares matrix: full	Hydrogen site location: inferred from neighbouring sites
*R*[*F*^2^ > 2σ(*F*^2^)] = 0.047	H-atom parameters constrained
*wR*(*F*^2^) = 0.143	*w* = 1/[σ^2^(*F*_o_^2^) + (0.0669*P*)^2^] where *P* = (*F*_o_^2^ + 2*F*_c_^2^)/3
*S* = 1.02	(Δ/σ)_max_ = 0.001
6315 reflections	Δρ_max_ = 0.58 e Å^−^^3^
583 parameters	Δρ_min_ = −0.36 e Å^−^^3^
Primary atom site location: structure-invariant direct methods	Extinction correction: none

### Fractional atomic coordinates and isotropic or equivalent isotropic displacement parameters (Å^2^)

**Table:**

	*x*	*y*	*z*	*U*_iso_*/*U*_eq_	Occ. (<1)
Cu1	0.46267 (7)	0.15476 (5)	0.36588 (4)	0.0406 (2)	
Cu2	−0.01499 (6)	0.64742 (5)	−0.11028 (4)	0.0407 (2)	
Cl1	0.87069 (17)	−0.11542 (12)	0.34408 (11)	0.0668 (5)	
Cl2	1.07017 (15)	−0.16022 (11)	0.69630 (11)	0.0578 (4)	
Cl3	0.39713 (16)	0.37698 (11)	−0.12877 (10)	0.0580 (4)	
Cl4	0.58637 (15)	0.33274 (11)	0.22575 (11)	0.0597 (4)	
N1	0.5002 (4)	0.1767 (3)	0.4841 (3)	0.0349 (10)	
N2	0.0214 (5)	0.6696 (4)	0.0075 (3)	0.0556 (13)	
O1	0.2819 (4)	0.2595 (3)	0.3616 (2)	0.0447 (9)	
O2	0.1508 (4)	0.3508 (3)	0.4497 (3)	0.0621 (12)	
O3	0.8857 (4)	0.3483 (4)	0.7126 (3)	0.0699 (12)	
H3	0.9506	0.3622	0.6795	0.105*	
O4	0.6311 (4)	0.0441 (3)	0.3785 (2)	0.0454 (9)	
O5	0.4122 (4)	0.1358 (3)	0.2489 (2)	0.0552 (10)	
H33	0.3333	0.1676	0.2174	0.083*	
H34	0.4672	0.1072	0.2129	0.083*	
O6	−0.1909 (4)	0.7570 (3)	−0.1170 (3)	0.0508 (10)	
O7	−0.3148 (4)	0.8569 (3)	−0.0328 (4)	0.0801 (15)	
O8	0.4986 (7)	0.8913 (6)	0.0835 (5)	0.051 (2)	0.507 (5)
H8	0.5564	0.8836	0.0429	0.077*	0.507 (5)
O8'	0.4246 (7)	0.9296 (6)	−0.1086 (6)	0.049 (2)	0.493 (5)
H8'	0.4912	0.9095	−0.0691	0.074*	0.493 (5)
O9	0.1554 (3)	0.5378 (3)	−0.0977 (2)	0.0459 (9)	
O10	−0.0682 (4)	0.6254 (3)	−0.2247 (2)	0.0547 (10)	
H35	−0.1283	0.6714	−0.2690	0.082*	
H36	−0.0357	0.5731	−0.2452	0.082*	
O11	0.8440 (5)	0.4610 (4)	0.3037 (3)	0.1056 (18)	
H37	0.8272	0.5183	0.2548	0.158*	
H38	0.8233	0.4217	0.2763	0.158*	
O12	0.8592 (8)	0.6491 (7)	0.3412 (6)	0.066 (3)	0.522 (10)
H39	0.8529	0.6666	0.3922	0.098*	0.522 (10)
H40	0.8643	0.5832	0.3637	0.098*	0.522 (10)
O13	0.4751 (9)	0.0597 (6)	0.7508 (5)	0.077 (3)	0.523 (6)
H41	0.4422	0.0976	0.7865	0.116*	0.523 (6)
H42	0.4076	0.0581	0.7184	0.116*	0.523 (6)
O12'	0.9281 (9)	0.5554 (7)	0.4185 (7)	0.066 (4)	0.478 (10)
H39'	0.8473	0.5526	0.4064	0.098*	0.478 (10)
H40'	0.9155	0.5923	0.4561	0.098*	0.478 (10)
O13'	0.3267 (10)	0.1743 (7)	0.8023 (6)	0.077 (3)	0.477 (6)
H41'	0.3386	0.1669	0.8620	0.116*	0.477 (6)
H42'	0.2932	0.1248	0.8019	0.116*	0.477 (6)
C1	0.2626 (6)	0.2932 (4)	0.4349 (4)	0.0468 (14)	
C2	0.3934 (5)	0.2649 (4)	0.5020 (4)	0.0404 (13)	
H2	0.3698	0.2435	0.5705	0.048*	
C3	0.4401 (5)	0.3661 (4)	0.4761 (4)	0.0456 (14)	
H3A	0.4656	0.3846	0.4087	0.055*	
H3B	0.3613	0.4241	0.4809	0.055*	
C4	0.5605 (5)	0.3567 (4)	0.5389 (4)	0.0380 (13)	
C5	0.5444 (6)	0.3550 (4)	0.6350 (4)	0.0486 (15)	
H5A	0.4579	0.3564	0.6614	0.058*	
C6	0.6528 (6)	0.3515 (4)	0.6913 (4)	0.0474 (14)	
H6	0.6388	0.3506	0.7551	0.057*	
C7	0.7826 (6)	0.3492 (4)	0.6543 (4)	0.0446 (14)	
C8	0.8034 (6)	0.3492 (4)	0.5601 (4)	0.0450 (14)	
H8A	0.8907	0.3471	0.5344	0.054*	
C9	0.6932 (6)	0.3523 (4)	0.5041 (4)	0.0429 (14)	
H9	0.7083	0.3513	0.4409	0.051*	
C10	0.6014 (5)	0.1182 (4)	0.5469 (4)	0.0380 (13)	
H10A	0.6016	0.1323	0.6050	0.046*	
C11	0.7153 (5)	0.0322 (4)	0.5356 (4)	0.0383 (12)	
C12	0.7253 (5)	0.0025 (4)	0.4506 (4)	0.0395 (13)	
C13	0.8490 (6)	−0.0788 (4)	0.4477 (4)	0.0452 (14)	
C14	0.9516 (5)	−0.1270 (4)	0.5211 (4)	0.0433 (14)	
H14	1.0306	−0.1798	0.5158	0.052*	
C15	0.9376 (5)	−0.0971 (4)	0.6025 (4)	0.0438 (14)	
C16	0.8219 (5)	−0.0183 (4)	0.6104 (4)	0.0411 (13)	
H16	0.8141	0.0017	0.6658	0.049*	
C17	−0.2082 (5)	0.7943 (4)	−0.0462 (4)	0.0420 (13)	
C18	−0.073 (2)	0.7774 (14)	0.0135 (14)	0.038 (4)	0.507 (5)
H18	−0.0932	0.7705	0.0814	0.045*	0.507 (5)
C19	−0.0263 (11)	0.8773 (8)	−0.0352 (8)	0.045 (3)	0.507 (5)
H19A	−0.0256	0.8894	−0.1047	0.053*	0.507 (5)
H19B	−0.0973	0.9373	−0.0259	0.053*	0.507 (5)
C20	0.1122 (19)	0.8796 (15)	−0.0029 (15)	0.042 (4)	0.507 (5)
C21	0.140 (2)	0.8750 (15)	0.0908 (16)	0.044 (4)	0.507 (5)
H21	0.0745	0.8647	0.1374	0.053*	0.507 (5)
C22	0.2662 (11)	0.8858 (8)	0.1162 (8)	0.046 (3)	0.507 (5)
H22	0.2784	0.8920	0.1767	0.055*	0.507 (5)
C23	0.3739 (14)	0.8875 (10)	0.0528 (10)	0.042 (3)	0.507 (5)
C24	0.347 (6)	0.887 (5)	−0.039 (5)	0.044 (9)	0.507 (5)
H24	0.4177	0.8887	−0.0834	0.053*	0.507 (5)
C25	0.218 (2)	0.8852 (13)	−0.0675 (12)	0.044 (4)	0.507 (5)
H25	0.2023	0.8875	−0.1307	0.053*	0.507 (5)
C18'	−0.101 (2)	0.7389 (15)	0.0366 (14)	0.036 (4)	0.493 (5)
H18'	−0.1411	0.6948	0.0915	0.043*	0.493 (5)
C19'	−0.0505 (10)	0.8163 (8)	0.0707 (8)	0.038 (3)	0.493 (5)
H19C	−0.1274	0.8811	0.0598	0.046*	0.493 (5)
H19D	−0.0285	0.7845	0.1405	0.046*	0.493 (5)
C20'	0.0742 (19)	0.8468 (15)	0.0232 (13)	0.032 (4)	0.493 (5)
C21'	0.0869 (12)	0.8771 (8)	−0.0768 (9)	0.040 (3)	0.493 (5)
H21'	0.0139	0.8811	−0.1167	0.048*	0.493 (5)
C22'	0.2054 (19)	0.9017 (13)	−0.1196 (11)	0.042 (4)	0.493 (5)
H22'	0.2126	0.9191	−0.1867	0.050*	0.493 (5)
C23'	0.313 (5)	0.900 (5)	−0.062 (5)	0.040 (8)	0.493 (5)
C24'	0.3069 (15)	0.8696 (10)	0.0395 (12)	0.042 (3)	0.493 (5)
H24'	0.3785	0.8676	0.0792	0.051*	0.493 (5)
C25'	0.188 (2)	0.8424 (17)	0.0773 (17)	0.046 (5)	0.493 (5)
H25'	0.1842	0.8192	0.1448	0.055*	0.493 (5)
C26	0.1228 (6)	0.6130 (5)	0.0709 (4)	0.0566 (16)	
H26	0.1228	0.6290	0.1280	0.068*	
C27	0.2371 (5)	0.5265 (4)	0.0609 (4)	0.0420 (13)	
C28	0.2481 (5)	0.4970 (4)	−0.0244 (4)	0.0371 (12)	
C29	0.3733 (5)	0.4155 (4)	−0.0259 (4)	0.0386 (13)	
C30	0.4740 (5)	0.3672 (4)	0.0480 (4)	0.0422 (13)	
H30	0.5542	0.3153	0.0431	0.051*	
C31	0.4552 (6)	0.3966 (4)	0.1310 (4)	0.0452 (14)	
C32	0.3407 (5)	0.4747 (4)	0.1372 (4)	0.0456 (14)	
H32	0.3307	0.4943	0.1928	0.055*	

### Atomic displacement parameters (Å^2^)

**Table:**

	*U*^11^	*U*^22^	*U*^33^	*U*^12^	*U*^13^	*U*^23^
Cu1	0.0356 (4)	0.0389 (4)	0.0449 (4)	−0.0035 (3)	−0.0044 (3)	−0.0167 (3)
Cu2	0.0304 (4)	0.0523 (4)	0.0438 (4)	−0.0084 (3)	0.0012 (3)	−0.0247 (3)
Cl1	0.0668 (11)	0.0601 (10)	0.0635 (10)	0.0104 (8)	−0.0035 (8)	−0.0319 (8)
Cl2	0.0417 (9)	0.0507 (9)	0.0644 (10)	−0.0008 (7)	−0.0143 (7)	−0.0070 (7)
Cl3	0.0579 (10)	0.0565 (9)	0.0624 (9)	−0.0029 (8)	0.0133 (8)	−0.0366 (8)
Cl4	0.0463 (9)	0.0514 (9)	0.0650 (10)	−0.0010 (7)	−0.0144 (7)	−0.0080 (7)
N1	0.032 (2)	0.029 (2)	0.042 (2)	−0.003 (2)	0.000 (2)	−0.014 (2)
N2	0.034 (3)	0.070 (3)	0.063 (3)	0.014 (2)	−0.008 (2)	−0.045 (3)
O1	0.034 (2)	0.049 (2)	0.051 (2)	−0.0024 (17)	−0.0083 (17)	−0.0244 (19)
O2	0.031 (2)	0.069 (3)	0.090 (3)	0.005 (2)	−0.010 (2)	−0.046 (2)
O3	0.053 (3)	0.105 (4)	0.062 (3)	−0.022 (3)	−0.009 (2)	−0.039 (3)
O4	0.044 (2)	0.041 (2)	0.049 (2)	0.0006 (17)	−0.0049 (19)	−0.0213 (18)
O5	0.045 (2)	0.066 (3)	0.053 (2)	0.002 (2)	−0.0085 (18)	−0.031 (2)
O6	0.043 (2)	0.052 (2)	0.060 (2)	−0.0029 (19)	−0.0139 (19)	−0.030 (2)
O7	0.033 (2)	0.079 (3)	0.144 (4)	0.008 (2)	−0.013 (3)	−0.075 (3)
O8	0.032 (4)	0.060 (5)	0.066 (5)	−0.006 (4)	−0.005 (4)	−0.033 (4)
O8'	0.026 (4)	0.054 (5)	0.067 (6)	−0.013 (4)	0.007 (4)	−0.021 (4)
O9	0.030 (2)	0.062 (2)	0.054 (2)	−0.0033 (18)	−0.0003 (18)	−0.039 (2)
O10	0.054 (3)	0.062 (3)	0.053 (2)	−0.007 (2)	−0.0147 (19)	−0.032 (2)
O11	0.103 (4)	0.138 (5)	0.078 (3)	−0.016 (4)	0.013 (3)	−0.057 (3)
O12	0.060 (6)	0.073 (7)	0.078 (7)	−0.020 (5)	0.012 (5)	−0.042 (6)
O13	0.090 (7)	0.076 (6)	0.069 (6)	−0.022 (5)	−0.004 (5)	−0.030 (5)
O12'	0.060 (6)	0.073 (8)	0.078 (7)	−0.020 (5)	0.012 (5)	−0.042 (6)
O13'	0.090 (8)	0.076 (7)	0.069 (6)	−0.022 (6)	−0.004 (5)	−0.030 (5)
C1	0.041 (4)	0.040 (3)	0.060 (4)	−0.009 (3)	−0.005 (3)	−0.020 (3)
C2	0.030 (3)	0.043 (3)	0.045 (3)	−0.002 (3)	−0.007 (2)	−0.017 (3)
C3	0.043 (3)	0.037 (3)	0.052 (3)	−0.001 (3)	−0.008 (3)	−0.017 (3)
C4	0.038 (3)	0.030 (3)	0.043 (3)	−0.002 (2)	−0.005 (3)	−0.016 (2)
C5	0.037 (3)	0.061 (4)	0.052 (3)	−0.014 (3)	0.010 (3)	−0.024 (3)
C6	0.047 (4)	0.064 (4)	0.034 (3)	−0.014 (3)	0.001 (3)	−0.021 (3)
C7	0.040 (3)	0.047 (3)	0.047 (3)	−0.005 (3)	−0.008 (3)	−0.020 (3)
C8	0.032 (3)	0.050 (3)	0.057 (4)	−0.006 (3)	0.004 (3)	−0.027 (3)
C9	0.048 (4)	0.044 (3)	0.040 (3)	−0.009 (3)	0.006 (3)	−0.023 (3)
C10	0.045 (3)	0.032 (3)	0.036 (3)	−0.008 (3)	−0.003 (3)	−0.012 (2)
C11	0.035 (3)	0.032 (3)	0.044 (3)	−0.006 (2)	−0.002 (2)	−0.011 (2)
C12	0.040 (3)	0.030 (3)	0.045 (3)	−0.008 (3)	0.000 (3)	−0.009 (2)
C13	0.049 (4)	0.035 (3)	0.050 (3)	−0.005 (3)	0.006 (3)	−0.017 (3)
C14	0.036 (3)	0.028 (3)	0.054 (3)	0.001 (2)	−0.002 (3)	−0.007 (3)
C15	0.028 (3)	0.036 (3)	0.055 (3)	−0.004 (3)	−0.006 (3)	−0.002 (3)
C16	0.042 (3)	0.036 (3)	0.046 (3)	−0.012 (3)	0.001 (3)	−0.013 (3)
C17	0.027 (3)	0.035 (3)	0.061 (4)	−0.007 (3)	0.000 (3)	−0.014 (3)
C18	0.027 (9)	0.041 (12)	0.047 (10)	−0.005 (7)	−0.002 (7)	−0.020 (8)
C19	0.032 (7)	0.043 (7)	0.061 (7)	−0.008 (5)	−0.008 (6)	−0.021 (6)
C20	0.031 (12)	0.039 (11)	0.054 (12)	−0.002 (7)	−0.002 (9)	−0.018 (8)
C21	0.032 (11)	0.048 (12)	0.053 (9)	−0.013 (9)	0.005 (7)	−0.017 (8)
C22	0.041 (7)	0.046 (7)	0.053 (7)	−0.011 (5)	0.003 (6)	−0.023 (6)
C23	0.034 (8)	0.040 (7)	0.054 (8)	−0.006 (6)	−0.002 (7)	−0.021 (6)
C24	0.04 (3)	0.040 (16)	0.06 (2)	−0.007 (17)	0.006 (15)	−0.022 (15)
C25	0.036 (9)	0.045 (9)	0.052 (10)	−0.004 (7)	−0.009 (11)	−0.023 (9)
C18'	0.024 (9)	0.038 (12)	0.047 (10)	−0.003 (7)	0.012 (6)	−0.022 (8)
C19'	0.032 (6)	0.040 (7)	0.045 (6)	−0.009 (5)	0.000 (5)	−0.019 (5)
C20'	0.024 (9)	0.036 (11)	0.042 (10)	−0.007 (6)	0.004 (7)	−0.021 (8)
C21'	0.030 (7)	0.042 (7)	0.048 (7)	−0.008 (5)	−0.003 (6)	−0.016 (6)
C22'	0.034 (8)	0.046 (9)	0.049 (9)	−0.009 (6)	−0.004 (9)	−0.021 (9)
C23'	0.03 (2)	0.039 (14)	0.05 (2)	−0.004 (15)	0.003 (14)	−0.022 (15)
C24'	0.034 (9)	0.042 (8)	0.049 (9)	−0.002 (7)	−0.004 (8)	−0.021 (6)
C25'	0.038 (13)	0.049 (12)	0.049 (9)	−0.005 (9)	0.000 (9)	−0.022 (8)
C26	0.040 (4)	0.076 (4)	0.060 (4)	0.005 (3)	−0.003 (3)	−0.048 (3)
C27	0.036 (3)	0.043 (3)	0.047 (3)	−0.001 (3)	0.006 (3)	−0.024 (3)
C28	0.028 (3)	0.043 (3)	0.046 (3)	−0.012 (3)	0.006 (3)	−0.022 (3)
C29	0.036 (3)	0.033 (3)	0.054 (3)	−0.011 (3)	0.015 (3)	−0.025 (3)
C30	0.036 (3)	0.026 (3)	0.060 (4)	−0.003 (2)	0.006 (3)	−0.014 (3)
C31	0.039 (3)	0.039 (3)	0.050 (3)	−0.008 (3)	−0.009 (3)	−0.008 (3)
C32	0.045 (4)	0.045 (3)	0.044 (3)	−0.003 (3)	−0.001 (3)	−0.020 (3)

### Geometric parameters (Å, °)

**Table:**

Cu1—O4	1.890 (3)	C8—C9	1.383 (7)
Cu1—N1	1.917 (4)	C8—H8A	0.9300
Cu1—O5	1.919 (3)	C9—H9	0.9300
Cu1—O1	1.953 (3)	C10—C11	1.441 (7)
Cu2—O9	1.896 (3)	C10—H10A	0.9300
Cu2—N2	1.909 (4)	C11—C16	1.406 (7)
Cu2—O10	1.915 (3)	C11—C12	1.422 (7)
Cu2—O6	1.946 (4)	C12—C13	1.423 (7)
Cl1—C13	1.733 (5)	C13—C14	1.366 (7)
Cl2—C15	1.756 (5)	C14—C15	1.371 (7)
Cl3—C29	1.738 (5)	C14—H14	0.9300
Cl4—C31	1.755 (5)	C15—C16	1.374 (7)
N1—C10	1.278 (6)	C16—H16	0.9300
N1—C2	1.463 (6)	C17—C18'	1.49 (2)
N2—C26	1.279 (6)	C17—C18	1.56 (2)
N2—C18'	1.47 (2)	C18—C19	1.527 (17)
N2—C18	1.55 (2)	C18—H18	0.9800
O1—C1	1.290 (6)	C19—C20	1.50 (2)
O2—C1	1.229 (6)	C19—H19A	0.9700
O3—C7	1.361 (6)	C19—H19B	0.9700
O3—H3	0.8200	C20—C21	1.38 (3)
O4—C12	1.301 (6)	C20—C25	1.39 (3)
O5—H33	0.8499	C21—C22	1.39 (3)
O5—H34	0.8498	C21—H21	0.9300
O6—C17	1.285 (6)	C22—C23	1.388 (18)
O7—C17	1.220 (6)	C22—H22	0.9300
O8—C23	1.366 (14)	C23—C24	1.38 (6)
O8—H8	0.8200	C24—C25	1.39 (5)
O8'—C23'	1.38 (7)	C24—H24	0.9300
O8'—H8'	0.8200	C25—H25	0.9300
O9—C28	1.303 (6)	C18'—C19'	1.54 (2)
O10—H35	0.8500	C18'—H18'	0.9800
O10—H36	0.8500	C19'—C20'	1.499 (18)
O11—H37	0.8504	C19'—H19C	0.9700
O11—H38	0.8502	C19'—H19D	0.9700
O12—H39	0.8501	C20'—C21'	1.39 (2)
O12—H40	0.8501	C20'—C25'	1.39 (3)
O13—H41	0.8500	C21'—C22'	1.39 (2)
O13—H42	0.8500	C21'—H21'	0.9300
O12'—H39'	0.8500	C22'—C23'	1.39 (4)
O12'—H40'	0.8500	C22'—H22'	0.9300
O13'—H41'	0.8500	C23'—C24'	1.39 (7)
O13'—H42'	0.8500	C24'—C25'	1.39 (3)
C1—C2	1.543 (7)	C24'—H24'	0.9300
C2—C3	1.532 (7)	C25'—H25'	0.9300
C2—H2	0.9800	C26—C27	1.442 (7)
C3—C4	1.494 (7)	C26—H26	0.9300
C3—H3A	0.9700	C27—C32	1.401 (7)
C3—H3B	0.9700	C27—C28	1.423 (7)
C4—C9	1.392 (7)	C28—C29	1.429 (7)
C4—C5	1.395 (7)	C29—C30	1.358 (7)
C5—C6	1.368 (7)	C30—C31	1.389 (7)
C5—H5A	0.9300	C30—H30	0.9300
C6—C7	1.379 (7)	C31—C32	1.354 (7)
C6—H6	0.9300	C32—H32	0.9300
C7—C8	1.379 (7)		
			
O4—Cu1—N1	94.31 (16)	C15—C16—H16	119.7
O4—Cu1—O5	88.98 (15)	C11—C16—H16	119.7
N1—Cu1—O5	176.21 (16)	O7—C17—O6	125.8 (5)
O4—Cu1—O1	174.80 (15)	O7—C17—C18'	116.7 (9)
N1—Cu1—O1	84.68 (15)	O6—C17—C18'	116.2 (9)
O5—Cu1—O1	91.87 (15)	O7—C17—C18	116.8 (9)
O9—Cu2—N2	94.16 (16)	O6—C17—C18	116.0 (9)
O9—Cu2—O10	89.46 (15)	C19—C18—N2	118.3 (12)
N2—Cu2—O10	174.82 (18)	C19—C18—C17	105.5 (12)
O9—Cu2—O6	177.43 (15)	N2—C18—C17	102.0 (11)
N2—Cu2—O6	83.96 (16)	C19—C18—H18	110.2
O10—Cu2—O6	92.31 (15)	N2—C18—H18	110.2
C10—N1—C2	120.7 (4)	C17—C18—H18	110.2
C10—N1—Cu1	125.7 (3)	C20—C19—C18	118.6 (13)
C2—N1—Cu1	113.5 (3)	C20—C19—H19A	107.7
C26—N2—C18'	118.4 (9)	C18—C19—H19A	107.7
C26—N2—C18	120.1 (8)	C20—C19—H19B	107.7
C26—N2—Cu2	126.6 (4)	C18—C19—H19B	107.7
C18'—N2—Cu2	112.6 (8)	H19A—C19—H19B	107.1
C18—N2—Cu2	112.7 (8)	C21—C20—C25	117.7 (17)
C1—O1—Cu1	114.9 (3)	C21—C20—C19	122.2 (17)
C7—O3—H3	109.5	C25—C20—C19	120.0 (17)
C12—O4—Cu1	127.0 (3)	C20—C21—C22	120.7 (17)
Cu1—O5—H33	124.3	C20—C21—H21	119.6
Cu1—O5—H34	126.9	C22—C21—H21	119.6
H33—O5—H34	107.4	C23—C22—C21	121.1 (14)
C17—O6—Cu2	115.6 (3)	C23—C22—H22	119.5
C23'—O8'—H8'	109.5	C21—C22—H22	119.5
C28—O9—Cu2	126.2 (3)	O8—C23—C24	123 (3)
Cu2—O10—H35	124.6	O8—C23—C22	118.9 (12)
Cu2—O10—H36	130.1	C24—C23—C22	118 (3)
H35—O10—H36	105.1	C23—C24—C25	121 (6)
H39—O12—H40	103.0	C23—C24—H24	119.4
H41—O13—H42	108.7	C25—C24—H24	119.4
H39'—O12'—H40'	105.2	C24—C25—C20	121 (4)
H41'—O13'—H42'	106.2	C24—C25—H25	119.4
O2—C1—O1	124.1 (5)	C20—C25—H25	119.4
O2—C1—C2	119.7 (5)	N2—C18'—C17	109.7 (13)
O1—C1—C2	116.0 (5)	N2—C18'—C19'	107.5 (12)
N1—C2—C3	112.2 (4)	C17—C18'—C19'	113.0 (13)
N1—C2—C1	108.1 (4)	N2—C18'—H18'	108.8
C3—C2—C1	106.6 (4)	C17—C18'—H18'	108.8
N1—C2—H2	109.9	C19'—C18'—H18'	108.8
C3—C2—H2	109.9	C20'—C19'—C18'	116.6 (12)
C1—C2—H2	109.9	C20'—C19'—H19C	108.1
C4—C3—C2	114.6 (4)	C18'—C19'—H19C	108.1
C4—C3—H3A	108.6	C20'—C19'—H19D	108.1
C2—C3—H3A	108.6	C18'—C19'—H19D	108.1
C4—C3—H3B	108.6	H19C—C19'—H19D	107.3
C2—C3—H3B	108.6	C21'—C20'—C25'	115.3 (15)
H3A—C3—H3B	107.6	C21'—C20'—C19'	122.7 (14)
C9—C4—C5	116.4 (5)	C25'—C20'—C19'	122.0 (16)
C9—C4—C3	122.0 (5)	C20'—C21'—C22'	122.1 (14)
C5—C4—C3	121.5 (5)	C20'—C21'—H21'	119.0
C6—C5—C4	121.7 (5)	C22'—C21'—H21'	119.0
C6—C5—H5A	119.2	C23'—C22'—C21'	120 (3)
C4—C5—H5A	119.2	C23'—C22'—H22'	120.2
C5—C6—C7	120.6 (5)	C21'—C22'—H22'	120.2
C5—C6—H6	119.7	O8'—C23'—C22'	117 (5)
C7—C6—H6	119.7	O8'—C23'—C24'	122 (3)
O3—C7—C8	122.3 (5)	C22'—C23'—C24'	121 (5)
O3—C7—C6	118.2 (5)	C25'—C24'—C23'	116 (3)
C8—C7—C6	119.6 (5)	C25'—C24'—H24'	122.0
C7—C8—C9	119.2 (5)	C23'—C24'—H24'	122.0
C7—C8—H8A	120.4	C24'—C25'—C20'	125.8 (19)
C9—C8—H8A	120.4	C24'—C25'—H25'	117.1
C8—C9—C4	122.4 (5)	C20'—C25'—H25'	117.1
C8—C9—H9	118.8	N2—C26—C27	125.2 (5)
C4—C9—H9	118.8	N2—C26—H26	117.4
N1—C10—C11	125.7 (5)	C27—C26—H26	117.4
N1—C10—H10A	117.2	C32—C27—C28	120.9 (5)
C11—C10—H10A	117.2	C32—C27—C26	117.3 (5)
C16—C11—C12	120.5 (5)	C28—C27—C26	121.7 (5)
C16—C11—C10	117.2 (5)	O9—C28—C27	125.5 (5)
C12—C11—C10	122.3 (5)	O9—C28—C29	119.9 (4)
O4—C12—C11	124.6 (5)	C27—C28—C29	114.5 (5)
O4—C12—C13	120.0 (5)	C30—C29—C28	123.9 (5)
C11—C12—C13	115.4 (5)	C30—C29—Cl3	118.6 (4)
C14—C13—C12	123.4 (5)	C28—C29—Cl3	117.6 (4)
C14—C13—Cl1	119.0 (4)	C29—C30—C31	119.0 (5)
C12—C13—Cl1	117.7 (4)	C29—C30—H30	120.5
C13—C14—C15	119.7 (5)	C31—C30—H30	120.5
C13—C14—H14	120.2	C32—C31—C30	120.7 (5)
C15—C14—H14	120.2	C32—C31—Cl4	121.0 (4)
C14—C15—C16	120.6 (5)	C30—C31—Cl4	118.2 (4)
C14—C15—Cl2	119.1 (4)	C31—C32—C27	120.9 (5)
C16—C15—Cl2	120.3 (4)	C31—C32—H32	119.6
C15—C16—C11	120.5 (5)	C27—C32—H32	119.6

### Hydrogen-bond geometry (Å, °)

**Table:**

*D*—H···*A*	*D*—H	H···*A*	*D*···*A*	*D*—H···*A*
O3—H3···O12^i^	0.82	1.88	2.643 (10)	154
O8—H8···O7^ii^	0.82	1.70	2.515 (9)	175
O5—H33···O6^iii^	0.85	1.89	2.714 (6)	164
O13—H42···Cl1^iv^	0.85	2.77	3.555 (9)	154
C26—H26···O3^v^	0.93	2.44	3.365 (7)	173

Symmetry codes: (i) −*x*+2, −*y*+1, −*z*+1; (ii) *x*+1, *y*, *z*; (iii) −*x*, −*y*+1, −*z*; (iv) −*x*+1, −*y*, −*z*+1; (v) −*x*+1, −*y*+1, −*z*+1.
